# An Examination of the Association between 5-HTTLPR, Combat Exposure, and PTSD Diagnosis among U.S. Veterans

**DOI:** 10.1371/journal.pone.0119998

**Published:** 2015-03-20

**Authors:** Yutao Liu, Melanie E. Garrett, Michelle F. Dennis, Kimberly T. Green, VA Mid-Atlantic MIRECC Registry Workgroup, Allison E. Ashley-Koch, Michael A. Hauser, Jean C. Beckham, Nathan A. Kimbrel

**Affiliations:** 1 Department of Cellular Biology and Anatomy, Georgia Regents University, Augusta, Georgia, United States of America; 2 Department of Medicine, Duke University Medical Center, Durham, North Carolina, United States of America; 3 Durham Veterans Affairs Medical Center, Durham, North Carolina, United States of America; 4 VA Mid-Atlantic Mental Illness Research, Education and Clinical Center, Durham, North Carolina, United States of America; 5 Department of Psychiatry and Behavioral Sciences, Duke University Medical Center, Durham, North Carolina, United States of America; Univ of Toledo, UNITED STATES

## Abstract

**Objective:**

To examine the association between the 5-HTTLPR polymorphism of the serotonin transporter (*SLC6A4*) gene, combat exposure, and posttraumatic stress disorder (PTSD) diagnosis and among two samples of combat-exposed veterans.

**Method:**

The first sample included 550 non-Hispanic Black (NHB) combat-exposed veterans. The second sample included 555 non-Hispanic White (NHW) combat-exposed veterans. Participants were genotyped for the 5-HTTLPR/rs25531 variants of the *SLC6A4* gene. A structured clinical interview was used to diagnose PTSD. Combat and civilian trauma exposure were assessed with validated self-report instruments. Logistic regression was used to test for main effects of 5-HTTLPR on PTSD diagnosis as well as gene x environment (GxE) interactions after adjusting for sex, ancestry proportion scores, civilian trauma exposure, and combat exposure.

**Results:**

Within the NHB sample, a significant additive effect was observed for 5-HTTLPR (*OR* = 1.502, *p* = .0025), such that the odds of having a current diagnosis of PTSD increased by 1.502 for each additional S’ allele. No evidence for an association between 5-HTTLPR and PTSD was observed in the NHW sample. In addition, no evidence for combat x 5-HTTLPR effects were observed in either sample.

**Conclusion:**

The present study suggests that there may be an association between 5-HTTLPR genotype and PTSD diagnosis among NHB veterans; however, no evidence for the hypothesized 5-HTTLPR x combat interaction was found.

## Introduction

Posttraumatic stress disorder (PTSD) is a complex disorder that develops following exposure to one or more traumatic events [1]. Although the majority of exposed individuals experience emotional distress immediately following a traumatic event, most do not go on to develop PTSD [1], which suggests that individual differences in genetic susceptibility might be at work [2]. This proposition is also supported by twin studies, which indicate that approximately 30% of the variability in PTSD risk is attributable to genetic factors (heritability) [3], [4], [5], [6].

Many genetic studies of PTSD to date have focused on the serotonin system, as PTSD can be treated by targeting the serotonin transporter (*SLC6A4*) with selective-serotonin reuptake inhibitors (SSRIs) [7], [8]. The extensively studied 5-HTTLPR variant of the *SLC6A4* gene is a repeat length polymorphism in the 5’ flanking promoter region that regulates gene expression levels [9]. This variant may contribute to variation in SSRI treatment response [10], [11]. To date, 5-HTTLPR has been investigated in more than 300 studies of neurological and psychiatric disorders, including PTSD [12], [13], [14], [15], [16], [17], [18], [19], [20], [21] major depressive disorder [22], and panic disorder [23].

Originally, 5-HTTLPR was analyzed as functionally biallelic with two major functional variants—long (L, 16 repeats) and short (S, 14 repeats) allele. The S allele leads to lower expression of 5-HTT mRNA and reduced serotonin transporter in membranes [24]. More recent studies have demonstrated that 5-HTTLPR, coupled with an A/G single-nucleotide polymorphism (SNP rs25531) that is observed almost exclusively in the L promoter variant (L_A_ and L_G_ alleles), is functionally triallelic [9]. Carriers of the S and L_G_ allele (genotypes L_G_/L_G_, S/L_G_, S/S) showed nearly equivalent 5-HTT expression levels, which were lower compared to the reference genotypes with average expression levels (S/L_A_, L_A_/L_G_). Moreover, relative to the same reference group, the homozygous L_A_/L_A_ genotype demonstrated approximately two-fold increased expression levels [9].

A number of previous investigations have examined the association between 5-HTTLPR and PTSD [12], [13], [14], [15], [16], [17], [18], [19], [20], [21]. For example, Wang and colleagues [19] examined the association between 5-HTTLPR and PTSD among a relatively small sample of U.S. combat veterans (*N* = 388) that was not stratified by race/ethnicity. This study found that that the S’ allele was associated with increased risk for a PTSD diagnosis among combat veterans. In contrast, two recent meta-analyses [20], [21] found no evidence for a direct association between 5-HTTLPR and PTSD across a variety of samples. Gressier and colleagues [20] did, however, note an association between 5-HTTLPR and PTSD among highly-traumatized populations, including veterans, leading them to suggest that there was a need for additional research aimed at examining the association between 5-HTTLPR, trauma severity, and PTSD risk among veterans and other highly traumatized populations. In addition, both meta-analyses [20], [21] concluded that there remained a significant need for additional gene x environment (GxE) research on the relationship between 5-HTTLPR and PTSD.

### Study Objectives

The present research had two primary objectives. Our first objective was to attempt to replicate Wang and colleagues’ [19] finding that S’ allele was associated with PTSD risk among combat veterans. Our second objective was to test the hypothesis that the S’ allele of the 5-HTTLPR polymorphism moderates the effect of combat exposure on PTSD risk among combat veterans. Whereas a number of studies have reported significant GxE effects for 5-HTTLPR on PTSD among civilian samples [12], [14], [15], [17], [18], no study to date that we are aware of has directly tested whether the S’ allele of the 5-HTTLPR polymorphism moderates the effect of combat exposure on PTSD risk among a large and well-characterized sample of combat veterans. However, as noted by Duncan and Keller [25] in their review of the first ten years of GxE interaction research in psychiatry, there is considerable evidence for a positive publication bias among novel GxE studies, highlighting the need for GxE replication studies that use similar methodology. Accordingly, the current study tested for both main effects and GxE effects using identical methods and statistical approaches in two well-characterized samples of combat veterans that were stratified by race and ethnicity.

## Method

### Participants and Procedures

Participants were recruited through fliers, advertisements and invitational letters to participate in one of several studies that assessed PTSD and collected a genetic sample, including the Mid-Atlantic Mental Illness Research, Education, and Clinical Center (MIRECC) Registry study and several independent trauma research studies conducted through the Durham VA Medical Center. All studies were conducted in compliance with the Declaration of Helsinki and received approval from the Durham VA Institutional Review Board prior to enrolling participants. After written informed consent was obtained, a structured diagnostic interview was administered to participants along with a battery of self-reports.

To be eligible for the current analyses, participants had to be: (1) a combat-exposed U.S. veteran, as defined by a score of 1 or higher on the Combat Exposure Scale (CES) [26]; (2) self-report their ancestry as either Non-Hispanic Black (NHB) or Non-Hispanic White (NHW); (3) have 5-HTTLPR genetic data available; (4) have trauma exposure data available; (5) have diagnostic data available; and (6) meet full diagnostic criteria to serve as either a case (i.e., the participant must have met all criteria for a current diagnosis of PTSD at the time of the assessment) or control (i.e., the participant must have never met lifetime diagnostic criteria for PTSD). Participants with other psychiatric diagnoses were included in the analyses in order to increase the generalizability of the findings. After the application of these inclusion/exclusion criteria and removal of any participants without 5-HTTLPR genetic data, a total of 550 NHB participants (77.8% males; 46.2% cases) and 555 NHW participants (90.6% males; 47.4% cases) were eligible to be included in the current analyses. Thus, the combined sample included a total 1105 veterans, 46.8% of whom met full Diagnostic and Statistical Manual for Mental Disorders—IV (DSM-IV) [27] criteria for PTSD at the time of the assessment.

### Phenotypic Measures

PTSD diagnoses were determined using either the Structured Clinical Interview for DSM-IV Disorders (SCID) [28] or the Clinician-Administered PTSD Scale for DSM-IV (CAPS) [29], depending upon which study the participant had originally participated in. PTSD was diagnosed with the SCID in 1019 participants (92.21%) from the MIRECC sample, whereas it was diagnosed with the CAPS in 23 participants (2.08%) from the other trauma-related studies. In addition, 63 participants (5.70%) from the MIRECC sample were missing the SCID. In these cases, scores ≥ 74 on the Davidson Trauma Scale (DTS) [30] were designated as PTSD cases.

Across studies, interviewers underwent extensive training, resulting in excellent reliability among clinical interviewers (Fleiss’ kappa = 0.94 among 22 interviewers). Interviewers also participated in ongoing supervision and monthly reliability meetings that included doctoral-level supervisors with expertise in the assessment and treatment of PTSD. A “current” diagnosis of PTSD (i.e., all DSM-IV diagnostic criteria were met at the time of the assessment) was used as the primary outcome variable in the current analyses.

The CES [26] is a widely-used 7-item, Likert-based scale designed to measure the level of wartime trauma exposure participants experienced during their deployments. The CES has good demonstrated good internal consistency and test-retest reliability in previous research with veterans [26]. Because combat exposure was an inclusion criterion for the current study, participants were required to score 1 or higher on the CES in order to be included in the current analyses. CES scores were also included as a covariate in the analyses in order to account for the potential influence of participants’ level of combat trauma on PTSD risk. CES scores were dichotomized into high and low combat using a cut-off score of 8 [26] in order to test for potential GxE interactions.

The Traumatic Life Events Questionnaire (TLEQ) [31] is a 23-item questionnaire designed to assess lifetime trauma exposure and response to traumatic events. For the purposes of the current study, the TLEQ total score was used as a covariate in the analyses in order to account for the potential influence of participants’ level of civilian trauma on PTSD risk. The TLEQ asks participant to report how many times they have experienced each of 22 different traumatic events. An additional TLEQ item provides participants with an opportunity to report a potentially traumatic event that the respondent did not feel comfortable specifying. Previous studies have demonstrated that the TLEQ is valid and reliable measure of lifetime trauma exposure [31], [32].

### Genotyping Methods

Genomic DNA was extracted from peripheral blood samples via alcohol and salt precipitation using Gentra Systems PUREGENE DNA Purification kit (Qiagen, Valencia, CA). Genotypes for 5-HTTLPR and rs25531 were combined into a triallelic polymorphism with the following alleles: S, L_A_, and L_G_. We developed a high throughput restriction fragment length polymorphism-based method that allows for determination of both variants (S/L; rs25531) with one assay. The 5-HTTLPR region was amplified by polymerase chain reaction using the oligonucleotide primers: 5'-/FAM/TGGCGTTGCCGCTCTGAATG-3', and 5'-AGGGACTGAGCTGGACAACCA resulting in PCR amplicons of length 528 for the L variant and 486 for the S variant. These fragments were then digested by restriction enzyme HpaII (New England Biolabs, Ipswich, MA), which cuts at the SNP rs25531 site only if the G allele is present. The digestion results in fragments of length 340bp corresponding to the L_A_ variant, 174bp for L_G_, and 297bp for the S variant. The digested PCR products were genotyped by size separation of the PCR fragment on the 3730 DNA Analyzer (Applied Biosystems, Foster City, CA) utilizing the 500LIZ Size Standard from Applied Biosystems. Of the resulting fragments from the digestion, only those from the FAM labeled end of the PCR product can be detected on the 3730. The raw data was examined in GeneMapper to determine the alleles for each sample. The L_A_ allele was detected at 340bp, L_G_ allele at 174bp, and S allele at 297bp. The observed sizes were consistent with the theoretical fragment sizes calculated using HpaII to digest the PCR product.

### Data Analysis Plan

Statistical analyses were performed using SAS software (version 9.4, SAS Institute Inc., Cary, NC). We tested for deviation from Hardy-Weinberg equilibrium (HWE) separately in cases and controls and by race/ethnicity group. To reduce the potential effects of population stratification, the NHB and NHW samples were analyzed separately. Principal components analysis (PCA) was run using the smartpca program from the software package EIGENSOFT [33]. Binary logistic regression was used so that the effects of 5-HTTLPR on PTSD could be examined after accounting for the influence of sex, ancestry, and trauma exposure. An additive genetic model (i.e., 0, 1 or 2 copies of the S’ allele) was used to estimate the main effect of 5-HTTLPR triallelic genotype on PTSD status in each of the samples. Sex, ancestry, lifetime civilian trauma exposure (TLEQ total score), and combat exposure (CES total score) were included as covariates. A second set of logistic regression models was also conducted to test for possible GxE effects between 5-HTTLPR and combat exposure. These models were identical to the first two models with two exceptions: (1) CES was dichotomized into high and low combat exposure using a cut score of 8 [26]; and (2) the 5-HTTLPR x combat exposure interaction term was also included in the model.

## Results

### Preliminary Analyses

Participant characteristics are provided in Table 1. None of the genotyped variants in the *SLC6A4* gene showed significant evidence for deviation from Hardy-Weinburg Equilibrium in PTSD cases or controls among either NHB or NHW (*p* > 0.05). As part of a larger ongoing genome-wide association study of PTSD, samples were genotyped on either the Illumina HumanHap650 Beadchip, Illumina Human1M-Duo Beadchip, or the Illumina HumanOmni2.5 Beadchip (Illumina, San Diego, CA). Data from the 1000Genomes project (www.1000genomes.org) was used to impute missing genotypes. PCA was then run on the NHB and NHW samples separately and scree plots were used to determine the appropriate number of principal components (PCs) needed to adequately control for population substructure in each subset. It was determined that three PCs were necessary for both the NHB and NHW subsets. In turn, these PCs were used in the logistic regression models to reduce the potential effects of population stratification.

**Table 1:** Participant characteristics.

	Non-Hispanic Black Veterans (*N* = 550)		Non-Hispanic White Veterans (*N* = 555)	
	Cases	Controls	Cases	Controls
Gender (% Male)	80.71%	75.34%	90.87%	90.41%
Age	38.37 (9.80)	38.67 (9.33)	34.49 (9.18)	36.76 (10.99)
Current PTSD	100%	0%	100%	0%
Lifetime PTSD	100%	0%	100%	0%
Lifetime Depression	65.47%	23.39%	64.50%	17.47%
Lifetime Bipolar Disorder	2.24%	0.34%	3.46%	0.68%
Lifetime Substance Use Disorder	56.95%	28.14%	59.31%	35.27%
Lifetime Schizophrenia	0%	0.34%	0%	0%
Lifetime Panic Disorder	6.28%	3.05%	8.23%	5.14%
Lifetime OCD	2.24%	1.69%	3.46%	0.34%
Lifetime Social Phobia	6.28%	3.05%	3.03%	2.40%
Lifetime Specific Phobia	5.83%	4.41%	3.46%	1.03%

*Note*: PTSD = posttraumatic stress disorder; OCD = obsessive-compulsive disorder.

### Logistic Regression Analyses

**Fig 1:**
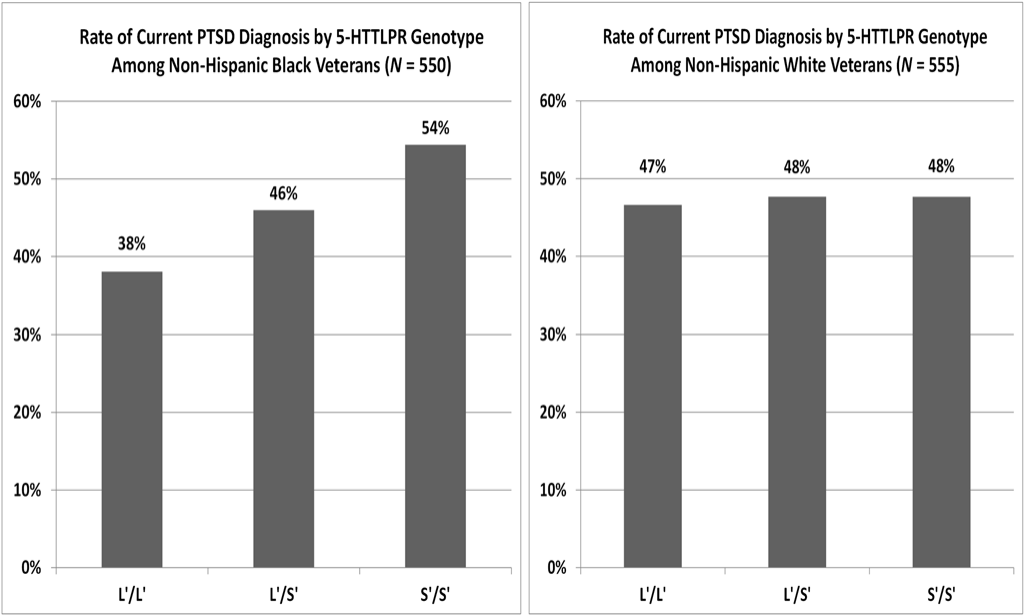
Rates of current PTSD diagnosis by 5-HTTLPR genotype.

A significant additive effect was observed for 5-HTTLPR (*OR* = 1.502, *p* = .0025) among NHB veterans, such that the odds of having a current diagnosis of PTSD increased by 1.502 for each additional S’ allele. As can be seen in Fig. 1, the rate of PTSD was higher among S’ homozygotes (87/160 or 54.38%) compared with heterozygotes (108/235 or 45.96%) and L’ homozygotes (59/155 or 38.06%). Civilian (*p* <. 0001) and combat trauma (*p* <. 0001) also had significant effects on risk for PTSD in the logistic regression model. Only one of the three PCs included in the model had a significant effect (*p* = .0177). Sex also had a non-significant effect (*p* = .69) on PTSD risk in the main effects logistic regression model.

A second logistic regression model was conducted on the NHB sample to test for potential 5-HTTLPR x combat exposure effects on PTSD status. The results from this model were highly similar to the first. 5-HTTLPR (*p* = .0025), civilian trauma (*p* <. 0001), combat trauma (*p* <. 0001), and PC1 (*p* = .0247) all continued to have significant main effects on PTSD; however, no other variable in the model, including the 5-HTTLPR x combat exposure interaction term (*p* = .65), was significant.

No main effect was observed for 5-HTTLPR (*p* = .4671) among NHW veterans. As can be seen in Fig. 1, the rate of PTSD among S’ homozygotes (71/149 or 47.65%), heterozygotes (123/258 or 47.67%), and L’ homozygotes (69/148 or 46.62%) was nearly identical. The only statistically significant predictors of PTSD status in the main effects model were civilian trauma exposure (*p* <. 0001) and combat trauma exposure (*p* <. 0001).

Similar results were obtained from the second logistic regression model that tested for 5-HTTLPR x combat exposure effects on PTSD. In this model, neither the 5-HTTLPR main effect (*p* = .24) nor the 5-HTTLPR x combat exposure interaction term (*p* = .51) was a significant predictor of PTSD status. Sex (*p* = .64) and the PCs (all *p’*s ≥. 22) also had non-significant effects on PTSD in this model as well. In contrast, both civilian (*p* <. 0001) and combat trauma (*p* = .0002) continued to have significant main effects in this model.

## Discussion

The finding of a main effect for the S’ allele of 5-HTTLPR on PTSD risk among NHB veterans provides partial support for our first hypothesis that the 5-HTTLPR S’ allele would be associated with increased risk for PTSD among combat veterans. We did not, however, find evidence for a main effect of 5-HTTLPR among NHW veterans. Thus, the findings from the present study represent only a partial replication of Wang and colleague’s [19] earlier study. One potential explanation for the discrepancy in findings between the current study and that of Wang et al. [19] is differences in analytical approach. Specifically, whereas Wang and colleagues [19] included both Black and White veterans in a single analysis, the present study examined NHB and NHW veterans separately in order to help to mitigate concerns about population stratification. The present study also included PCs in the logistic regression models to help to account for population substructure. Thus, it is possible that the different analytical approaches may have contributed to the differences in findings between our study and that of Wang et al. [19]. Another explanation for the findings from the present research is that race/ethnicity may moderate the effect of 5-HTTLPR on PTSD among combat veterans; however, since the majority of participants in Wang et al.’s study were White, whereas we only find evidence for an association among NHB (and not NHW) veterans, the latter explanation seems unlikely.

Regarding our second hypothesis, we found no evidence that 5-HTLLPR moderates the effect of combat exposure on PTSD among either sample of combat veterans. While the current study is the first study that we are aware of to directly test this hypothesis among two well-characterized samples of combat veterans, this finding contrasts with several prior studies finding evidence for GxE effects for 5-HTTLPR on PTSD among civilian samples [12], [14], [15], [17], [18]. Thus, it may be the case that the GxE effects reported in previous studies were unique to the specific types of traumatic and stressful experiences studied among these groups of traumatized civilians. Since the current study does not represent a direct replication of these studies, there remains a need for additional work aimed at attempting to replicate these studies directly [25]. Moreover, while the findings from the present research indicate that 5-HTTLPR may not interact with combat exposure to predict PTSD, it should be noted that there is other recent evidence of combat x gene effects on PTSD from both twin studies [34] and studies of other candidate genes, such as apolipoprotein E (APOE) [35], [36]. Thus, additional research on combat x gene effects on PTSD appears warranted. Such research is especially needed among samples of Iraq/Afghanistan veterans, where rates of PTSD appear to be particularly high [37], [38].

### Strengths and Limitations of the Present Research

Strengths of the current study include: (1) the use of a combat-exposed control group with no current or lifetime PTSD, (2) the inclusion of two ethnically diverse groups of veterans, (3) separate analysis of the NHB and NHW samples as well as the use of PCs to help to account for population stratification, and (4) a well-defined clinical phenotype. Limitations of the current study include: (1) a relatively small sample size for psychiatric genetics research (particularly GxE research), (2) the use of DSM-IV [27] criteria to define PTSD, and (3) failure to capture clinician-rated symptom severity ratings on all of the participants. Regarding the latter point, because only a small portion of the sample completed the CAPS (Blake et al., 1995) as part of the non-MIRECC studies, we did not have adequate statistical power to test for 5-HTTLPR effects on clinician-rated symptom severity in the current study. Thus, additional research on the potential relationship between 5-HTTLPR, combat exposure, and clinician-rated PTSD symptom severity among combat veterans is still needed.
